# Epidemiology of enuresis: a large number of children at risk of low regard

**DOI:** 10.1186/s13052-020-00896-3

**Published:** 2020-09-11

**Authors:** Pietro Ferrara, Giulia Franceschini, Federico Bianchi Di Castelbianco, Rachele Bombace, Alberto Villani, Giovanni Corsello

**Affiliations:** 1 Institute of Pediatrics, Catholic University Medical School, Largo A. Gemelli, 8, 00168 Rome, Italy; 2 Service of Pediatrics, Campus Bio-Medico University, Rome, Italy; 3 Istituto di Ortofonologia (IdO), Rome, Italy; 4 Agenzia DIRE, Rome, Italy; 5 Pediatric and Infectious Disease Unit, Bambino Gesù Children’s Hospital, IRCCS, Rome, Italy; 6 Neonatal Intensive Care Unit, AOUP “P. Giaccone”, Department of Health Promotion, Mother and Child Care, Internal Medicine and Medical Specialties “G. D’Alessandro”, University of Palermo, Palermo, Italy

**Keywords:** Enuresis, Children, Epidemiology

## Abstract

**Aim:**

To describe the epidemiological aspects of nocturnal enuresis (NE). In this study we identify the prevalence and the familial conditions in a large, representative sample of children with monosymptomatic NE (MNE) and nonmonosyptomatic NE (NMNE).

**Material and methods:**

In this descriptive-analytic study the Italian Society of Pediatrics (SIP) promoted a prevalence study of NE using a questionnaire involved 320 primary care Pediatricians from Northern, Central and Southern Italy, from January 2019 to July 2019, with a total of 130,000 children analyzed by questionnaire related to epidemiology and type of NE, familiarity, quality of sleep, eating and drinking habits, pharmacological and psychological/behavioural interventions and family involvement.

**Results:**

270/320 (84.4%) Paediatrician replied to our questionnaire. We enrolled a total of 9307/130,000 (7.2%) children with NE, aged between 5 and 14 years: 2141 diagnosed with MNE and 7176 qualified as NMNE. Poor quality of sleep were reported in 7064 patients; 90% of children did not consider a dietary and drinking recommendation. Pediatrician reported a total of 54.1% of parents who declared to have a negative reaction to their children because of the bedwetting. A percentage of 71.4% of parents declared to use or to have used alternative therapies and not to prefer, at first, a pharmacological intervention.

**Conclusion:**

The choice of treatment should include psychological/behavioural interventions in all cases to improve the therapeutic outcome. All primary care Pediatricians should be aware of the all aspects of NE to choose the best way to treat every patient.

## Introduction

Nocturnal enuresis (NE) is a common condition in children that can cause low quality of life to the child and to his/her family. NE is defined as an intermittent (i.e., not continuous) bedwetting with any frequency while sleeping in children, according to the International Children’s Continence Society (ICCS) [1]. NE in children without any other lower urinary tract (LUT) symptoms and without a history of bladder dysfunction is defined as monosymptomatic NE (MNE). Children with concomitant daytime incontinence is still called NE (or nocturnal incontinence), although it belongs to the nonmonosymptomatic variety (NMNE).

Three major pathogenetic mechanisms have been established as essential and they are nocturnal polyuria, detrusor overactivity and associated with failure to awaken in response to bladder sensations. Each theory can be supported by various studies, but no one is reported to explain bedwetting in all children [2].

The prevalence of NE is variable. It is above 10% among 6 year-old, around 5% among 10 year-old, and 0.5–1% among teenagers and young adults [3–5].

According to the document of the International Children’s Continence Society (ICCS) psychopathology is important too: 20–30% of the children with NE have at least one psychological/psychiatric disorder at rates two times higher than non-wetting children [6]. It may be linked to numerous negative psychosocial consequences, including lower health related quality of life, increased behavioral problems and impaired neuropsychological functioning.

Despite the high prevalence of NE, doctors in a primary care setting may have difficulty in the evaluation and management of this condition. Once comprehensive history taking and evaluation of daytime symptoms or comorbidities, MNE can be managed efficaciously in the majority of cases.

The choice of treatment depends on the frequency and severity of symptoms, the child’s age and motivation; even spontaneous remission may be frequent, but management of NE should be carried out because of its deep impact on a child’s emotional state, self-esteem, families, social daily life and global quality of life.

In this study we aimed to describe the different epidemiological aspects of NE in children from Italy analysing prevalence and type of NE, comorbidity, therapeutic choices, pharmacological and psychological/behavioural interventions, dietary associated factors in samples collected from Italian Pediatricians.

## Material and methods

In this retrospective-descriptive-analytic survey the Italian Society of Pediatrics (SIP) promoted a prevalence study of NE using a questionnaire and enrolling 320 primary care Pediatricians from Northern, Central and Southern Italy. From January 2019 to July 2019, SIP invited Pediatricians to fill out a questionnaire made up of inquiries about their patients and NE. The questionnaire was assessed for content validity and modified accordingly by several experts and based on international position papers. Pediatrician took part into the survey by providing data from their catchment area and using their medical data base, with a total pediatric population amounts to 130,000 children. An anonymous questionnaire without the need for informed consent, was administered via an application that collected the data automatically by Agenzia DIRE. The raised questions consisted of a set of questions related to epidemiology and type of NE, familiarity, quality of sleep, eating and drinking habits, pharmacological and psychological/behavioural interventions, family involvement and management of the condition by parents. All procedures involving human participants were performed in accordance with the ethics standards of the institutional and/or national research committee, and in accordance with the 1964 Declaration of Helsinki, its later amendments, or comparable ethical standards and was approved by the Institutional Review Board of Italian Society of Pediatrics. Data were analysed using SPSS V.24.0 (SPSS) for windows.

## Results

**Fig. 1:**
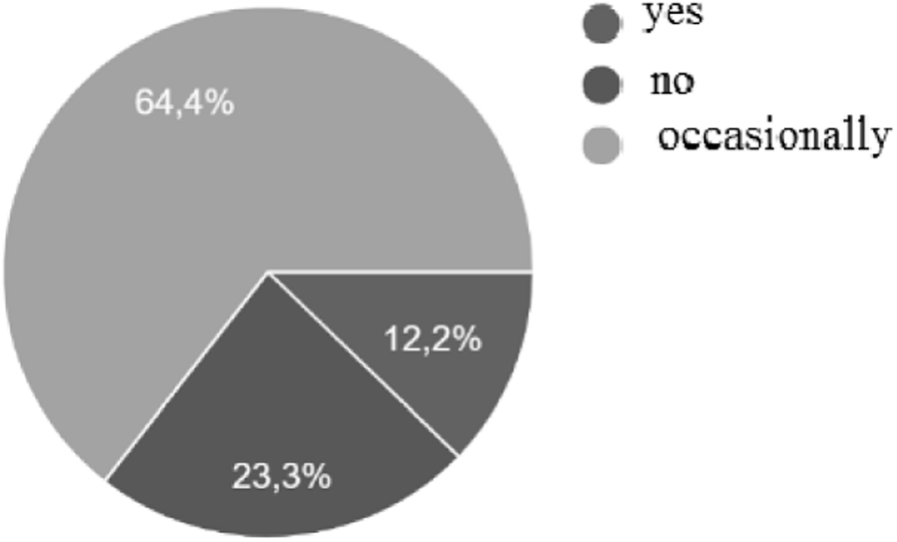
Percentage of patients with daytime symptoms

Out of 320 Paediatrician involved, 270 caring for 130,000 patients, were returned with a response rate of 84.4%. We were able to examine a sample of 130,000 children. A total of 9307/130,000 (7.2%) children, aged between 5 and 14 years (mean age 8.32 ± 2.45 years) presented NE with a family history positive for NE in 4185/9307 (44,9%)(Table 1). In the study 5934 males and 3373 females were included. Among 9037 patients with a documented history of NE, 2141 (23%) were diagnosed with MNE and 7166 (77%) were qualified as NMNE continuous or sporadic with one or more of the following symptoms: daytime incontinence, dysuria, urgency, holding manoeuvres, and urgency incontinence (Fig. 1).

**Table 1:** Incidence rate per age group

Years	n (%)
5–6	1983 (21.3%)
7–8	2393 (25.7%)
9–10	1814 (19.5%)
11–12	1684 (18.1%)
> 12	1433 (15.4%)

**Fig. 2:**
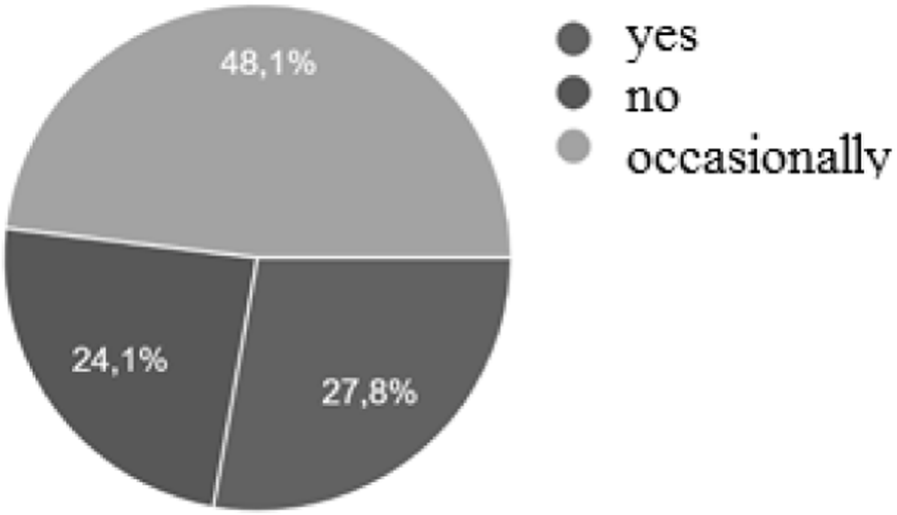
Percentage of patients with poor quality of sleep

Poor quality of sleep, strongly associated with NE, were reported in 7064 patients (75,8%): in a percentage of 27.8% every night and of 48.2% occasionally (Fig. 2) (Table 2).

**Table 2:** Characterisctics of 9307 enuretic patients

Mean age	8.32 ± 2.45 years
Male	5934 (63,7%)
Female	3373 (36,3%)
Family history of NE	4185 (44,9%)
Poor quality of sleep	7064 (75,8%)
MNE	2141 (23%)
NMNE	7166 (77%)

By analysing the eating and drinking habits, we underlined that in 90% of cases, children did not consider a drinking recommendation: liquids in 40% of cases were taken up to 8 p.m.; in 60% of cases patients had no time limits even though all Pediatricians recommended not to drink water after 8 p.m. Children clamed to drink mainly water, 9% also reported juices and another 9% soft drinks too. As regard the type of diet, Pediatricians reported a balanced diet for their patients with fruit and vegetables included**.** A percentage of 20% of Pediatricians reported high-protein and/or lipid-rich and/or low-fiber diets in their patients.

Pediatricians analysed from their records the social and educational status of the families and the parent’s declarations and they found that a total of 54.1% of parents declared to react negatively to their children because of the bedwetting such as leaving wet bed, sleep deprivation and disciplinary measures such as humiliation.

**Fig. 3:**
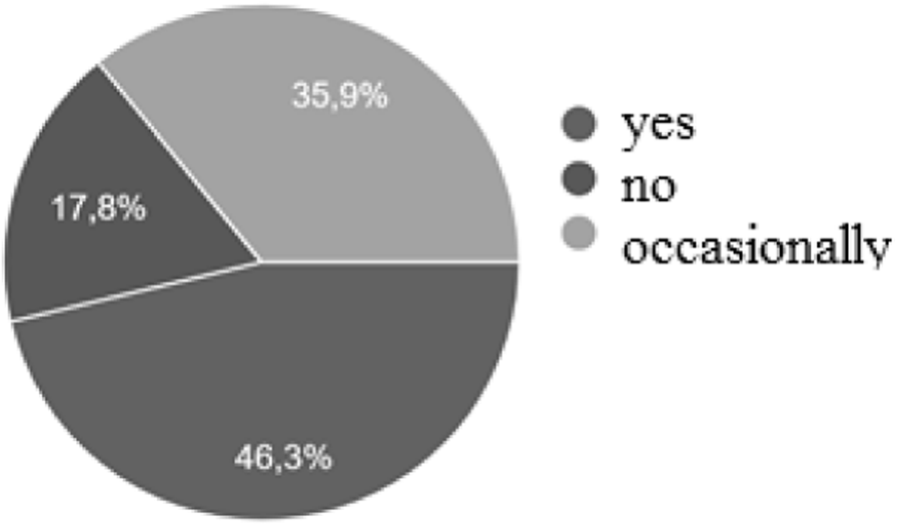
Percentage of school performance improvement

Parents declared a children school performance improvement, alongside the decrease of number of wet bed night, in a 82.2% of cases (Fig. 3).

Lastly, a percentage of 71.4% of parents declared to use or to have used alternative therapies such as homotoxicological remedies, acupuncture, simple behavioural interventions, and not to prefer, at first, a pharmacological intervention.

## Discussion

Our findings suggest that NE affect a significant number of children and adolescents but it is still underestimated. Over 130,000 children analyzed by questionnaires, the prevalence of NE was 7.1% and only 25% of this population suffer from MNE. The first interesting consideration is that most cases the diagnosis in NMNE (75%). The diagnosis of NMNE is essentially based on a thorough evaluation of the patient’s clinical history and clinical examination investigating daytime symptoms. We confirmed family history positive in a high percentage of cases.

NE is often associated with specific comorbidities, such as urinary tract infections and fecal incontinence with or without constipation, especially in NMNE. Moreover there are psychiatric and behavioral disorders associated with neurobehavioral externalizing conditions such as attention deficit hyperactivity disorder. Many studies have demonstrated the etiopathogenesis of these associations and others will be necessary to confirm it and to show that the treatment of comorbidities improves symptoms of voiding disorders. A study, by Ferrara et al. in 2019 based on a national population database shown a strong family history in children with NE [7]. NE was heredity in the 31.2% of cases. Were found urogenital abnormalities in 15.7% of cases, innocence heart murmur in 21.4% of cases and parasomnias in a good percentage of cases, especially snoring (13.7%), restless sleep (5.7%), somniloquy (23.7%) and bruxism (14.7%). In N-MNE group, 81% of boys and 93.8% of girls have deep sleep. Moreover neurobehavioral conditions were associated in 5.7% of patients. Cutaneous manifestations of spinal dysraphism, such as pilonidal dimple, single and deflected intergluteal cleft, or double intergluteal cleft were found in 5.2% patients. Encopresis was found in 3% children and 14.5% suffered from constipation. In 7.7% children was found polythelia. These data suggested that deep sleep is more frequent in NE children vs nonenuretic children as well as snoring, urological abnormalities, innocent heart murmur, constipation, spinal dysraphism and polytelia.

A further important point shown in the study concerns the behaviour of parents: indeed we underlined how punishment because of NE is applied to 12.4% of children. This analysis brings out an increased number with 54.1% of parents who declared to punish their children because of the bedwetting. In particular we point out those following type of punishment: reproaches in most cases, than sleep deprivation, disciplinary measures, humiliation and corporal punishment, without realizing it. Parents should be sensitized on the adverse effects of punishment on child development. For this reason it is important to explain the definitions of the disorder and find the best treatment (behavioural and/or pharmacological) depending on the single patient, his/her family and compliance of both. Family quality of life is compromised; for this reason family involvement and management of the condition by parents have to be considered too [8].

Moreover sleep disordered or poor quality of sleep in children is linked to numerous negative psychosocial consequences, including lower health related quality of life, increased behavioural problems impaired neuropsychological functioning and lower school performance [9]. Decreasing the number of wet bed nights, in fact, we underline a children school performance improvement, in a 82.2% of cases.

Many studies show that some foods and beverages can promote diuresis or bladder irritability, which in some people can exacerbate bladder symptoms and NE and compared the efficacy of combined specific dietary advices and dDAVP vs dDAVP alone [10]. For this reason we analyzed the eating and drinking habits, but we underlined that in 90% of cases, children did not consider a dietary and drinking recommendation even though all Pediatricians recommended not to drink water after 8 p.m. Children declared to drink mainly water, but also juices and soft drinks, that can decrease the efficacy of therapy. We highlight, on the upside, the crucial importance of dietary advices.

The choice of treatment depends on the frequency and severity of symptoms, the child’s age and motivation; we want to underline that the parents educational level may influence the choice of treatment such as low educational level, working mothers, jobless and crowded family [11].

Severity of NE was classified as mild-moderate and severe according to the frequency of NE. More than 5 wet nights weekly was classified as severe and 5 or fewer as mild-moderate. Moreover association between NE and specific comorbidities such as sleep disorders, encopresis, headache, and psychiatric and behavioral disorders, can worse the clinical pattern [12].

A large percentage of parents declared to use or to have used alternative therapies and not to prefer, at first, a pharmacological intervention; this data is found in the literature too, where systematic reviews are presented to declare the possible effectiveness and safety of the following alternative interventions such as homotoxicology, acupuncture, dry bed training, enuresis alarm, and hypnotherapy [13, 14]. Scientific evidence had underlined the primary focus is on non-invasive urotherapy, which contains many elements of cognitive behavioural therapy that can be accompanied by targeted pharmacotherapy too [15, 16].

## Conclusions

NE is a very common disorders with high prevalence in Italy and a deep impact on children and families quality of life. Enuretic children experience some kind of punishment, nonetheless its involuntary nature. Pediatrician should explain that no punishment would help improve the condition; all families should be sensitized on the adverse effects of punishment on child development in general. The choice of treatment should include psychological/behavioural interventions in all cases to improve the therapeutic outcome.

All Pediatricians should be aware of the all aspects of NE to choose the best way to treat every patient on reducing the number of wet nights as well as psychological aspects of the condition.

Further studies should focus on the prognostic role of comorbidities associated with NE.

## Supplementary information

**Additional file 1.**

## Data Availability

The datasets used and/or analysed during the current study are available from the corresponding author on reasonable request.

## Abbreviations

NE: Nocturnal enuresis
MNE: Monosymptomatic nocturnal
NMNE: Nonmonosyptomatic nocturnal enuresis
SIP: Italian Society of Pediatrics
ICCS: International Children’s Continence Society
