# Supplementary material for: Epidemiology of enuresis: a large number of children at risk of low regard
Source: Ital J Pediatr. 2020 Sep 11;46:128. doi: 10.1186/s13052-020-00896-3 (PMC7488742; doi:10.1186/s13052-020-00896-3)
Supplement: Supplementary file 1 — Additional file 1. [file 13052_2020_896_MOESM1_ESM.docx]

Dear Editor,

thank you for considering our manuscript for publication. Moreover, we would like to thank reviewers to have shown interest in our manuscript and for their comments on it.

We revised it and all the changes are highlighted in yellow.

**Answers to Comment:**

**Reviewer**

Thank you for opinion

**Q** [..] As mentioned in your added content, NE was usually associated with some specific factors such as family history, poor quality of sleep and so on. These risk factors of NE should be analyzed statistically based on your own results to find their relevance to NE.

**R.** *We added as suggested the following text in the section Discussion.*

“NE was heredity in the 31.2% of cases. We found urogenital abnormalities in 15.7% of cases, innocence heart murmur in 21.4% of cases and parasomnias in a good percentage of cases, especially snoring (13.7%), restless sleep (5.7%), somniloquy (23.7%) and bruxism (14.7%). In N-MNE group, 81% of boys and 93.8% of girls have deep sleep. Moreover neurobehavioral	conditions	were	associated	in 5.7% of patients. Cutaneous manifestations of spinal dysraphism, such as pilonidal dimple, single and deflected intergluteal cleft, or double intergluteal cleft were found in 5.2% patients. Encopresis was found in 3% children and 14.5% suffered from constipation. In 7.7% children was found polythelia. These data suggested that deep sleep is more frequent in NE children vs nonenuretic children as well as snoring, innocent heart murmur, spinal dysraphism and polytelia.”
